# The value of negative stress echocardiography in predicting cardiovascular events among adults with no known coronary disease

**DOI:** 10.15171/jcvtr.2019.16

**Published:** 2019-06-13

**Authors:** Niloufar Samiei, Mozhgan Parsaee, Leili Pourafkari, Arezou Tajlil, Yeganeh Pasbani, Ali Rafati, Nader D Nader

**Affiliations:** ^1^Heart Valve Research Center, Rajaie Cardiovascular Medical & Research Center, Tehran, Iran; ^2^Echocardiography Research Center, Rajaie Cardiovascular Medical & Research Center, Tehran, Iran; ^3^Department of Anesthesiology, State University of New York at Buffalo, Buffalo, NY, USA

**Keywords:** Stress Echocardiography, Long-term Mortality, Revascularization, Coronary Artery Diseases, Prognostic Factors

## Abstract

***Introduction:*** Stress echocardiography is a safe and cost-effective method of evaluating the patients with suspected coronary artery disease (CAD). However, the risk factors of an adverse cardiovascular event after a normal exercise (ESE) or dobutamine (DSE) stress echocardiography are not well established.

***Methods:*** A cohort of 705 patients without previous history of CAD and a negative ESE/DSE was studied. All studies were performed in a high-volume echocardiologic laboratory and interpreted by two experienced echocardiography-trained cardiologists. Patients with inconclusive studies and those with an evidence of myocardial ischemia were excluded. Demographic, echocardiographic and hemodynamic findings were recorded. Patients were followed for at least 2 years. Independent predictors of major adverse cardiovascular events (MACE) were determined by regression analysis.

***Results:*** During a period of 55.7±17.5 months, MACE occurred in 35 (5.0%) of patients. Negative predictive value (NPV) of DSE was 89.2%, which was significantly less than 96.5% for ESE in predicting the occurrence of MACE (*P *= 0.001). MACE occurred more frequently among older (≥65 years) men with preexisting diabetes, hypertension, and/or hyperlipidemia. During ESE, a higher maximum blood pressure*heart rate product for the achieved level of metabolic equivalent (METS) of tasks was also an independent predictor of MACE.

***Conclusion:*** Inability of patients to undergo traditional ESE that led to the choice of using DSE alternative reduces the NPV of the stress echocardiography among patients without previous history of CAD. A modest rise of heart rate and blood pressure in response to increased level of activity serves as favorable prognostic value and improves the NPV of stress echocardiography.

## Introduction

Coronary artery diseases (CAD) are the leading cause of mortality and morbidity worldwide. For optimizing the treatment and improving the prognosis, diagnosing CAD in individuals at risk or in patients with symptoms suggestive of a possible CAD is essential.^1^ Among the available modalities for diagnosing CAD in either ambulatory or emergency settings, stress echocardiography is proven as a cost-effective and safe functional test in appropriately selected patients.^2-6^ Even though stress echocardiography has a high negative predictive value (NPV),^7^ the factors that predict a worse outcome in the setting of a normal study are not well-defined.

In addition to its diagnostic value, the echocardiographic findings and stress-induced changes in those variables have been previously used for risk stratification of patients with a possibility of CAD.^4,6,8^ Reduced functional capacity, left ventricular dysfunction and higher peak wall motion score index during stress echocardiography can be indicative of less favorable prognosis.^9-11^ Though, these factors make the test positive and do not apply to patients with a normal study. While having a high NPV helps clinicians to avoid further diagnostic interventions,^7^ it is essential to identify the patients with lower event-free survival after a normal stress echocardiography exam.

We followed up all patients with no history of CAD who had a normal exercise (ESE) or dobutamine (DSE) stress echocardiography for at least 2 years. Main objective was to evaluate the predictive value of a stress echocardiography in the occurrence of major cardiac event this population. We hypothesized that a constellation of patient risk factors and echocardiographic parameters could help in predicting development of adverse events after a normal stress echocardiography study in a population with no known history of CAD.

## Materials and Methods

This was a cohort follow-up study of patients with no previous history of CAD and symptoms of atypical chest pain, unexplained dyspnea or palpitation who were referred for an evidence of cardiac ischemia and were found to have a negative stress echocardiography (ESE/DSE) from August 2012 to February 2017. The study protocol was reviewed and approved by the institutional review board of our university. All patients signed a privacy of health information authorization for research while they signed an informed consent for the procedure. Patient privacy was maintained in all study process. All studies were performed in the echocardiography laboratory a high-volume cardiac center and were interpreted as negative for inducible ischemia by two subspecialty-trained cardiologists in echocardiography.

Patients with a known history of CAD, systolic heart failure with reduced left ventricular ejection fraction, percutaneous coronary angiography or coronary artery bypass graft surgery prior to the stress echocardiography were all excluded from this study. Additionally, those with more than moderate valvular dysfunction, history of permanent pacemaker implantation and non-sinus rhythm also exclusion criteria were not included. All demographic information, cardiovascular risk factors, echocardiographic findings were retrospectively reviewed and recorded in the prepared datasheets. Follow-up data were collected prospectively by contacting patients by phone and/or electronic hospital record. Patients without at least two years follow-up from the index stress echocardiography exam were excluded from the study.

### Stress echocardiography

Patients underwent treadmill ESE or pharmacological stress echocardiography with Dobutamine based on established standard protocols. The preferred method of stress echocardiography was ESE except in patients who were not able to exercise in whom DSE was performed. Treadmill ESE was performed using the standard Bruce protocol^12^ for the achieved metabolic equivalent of tasks (METS) activity levels. DSE was performed by continuous intravenous infusion of dobutamine in 3-minute intervals, starting with 5 μg/kg/min and then increasing to 10, 20, 30, and 40 μg/kg/min. In case 85% of maximal heart rate was not achieved, Atropine, in divided doses of 0.25 mg to 0.5 mg to a total of 2.0 mg was added. A normal exam was defined as normokinetic segments at rest and normal or hyperkinetic segments during stress in patients who achieved 85% of maximal heart rate.^13^

### Study variables and endpoints

Demographic information including age at the time of stress echocardiography and sex, as well as cardiovascular risk factors including diabetes mellitus, current smoking, hypertension, hyperlipidemia and family history of premature coronary heart diseases, were collected from medical records for each patient. The presenting symptoms at the time of referral to the echocardiography laboratory were also recorded. Baseline echocardiographic variables, as well as stress-induced echocardiographic findings were 2-level verified by board certified experienced level III cardiologists,^14^ and the data was gathered for each patient. Peak rate-pressure product (RPP_max_) was calculated as the product of peak heart rate (HR_max_) and peak systolic blood pressure (SBP_max_) in centimeters of mercury. The primary endpoint was the occurrence of a composite cardiovascular event including all-cause death, acute coronary syndrome, diagnostic coronary angiography, percutaneous or surgical coronary revascularization, cerebrovascular accidents or any hospital admission due to cardiac causes during the follow-up period. We also recorded the date of the event according to the patients’ recollection. The date of the phone call follow-up was entered for all patients who did not experience any event for time-to-event analysis. The secondary outcome variables were the occurrence of any individual event.

### Statistical analysis

Data were analyzed by SPSS Statistics for Mac OS; version 25.0 (IBM Inc., Chicago, IL). Cross-tabulation with chi-square analyses/Fishers exact test were used to analysis the frequency of all categorical variables against the frequency of the primary end-point and the results were presented as frequencies and percentages. The one-dimensional probability distribution of the continuous variables was first examined with Kolmogorov-Smirnov goodness-of-fit test. Variables with a normal distribution were compared using independent t-tests and presented as mean ± standard variation. Variables which failed a normality test were analyzed using a non-parametric U-test and presented as median (interquartile range-IQR). Kaplan–Meier analysis with log-rank statistics were used for comparisons of major adverse cardiovascular events (MACE) between study variables. Cox multivariate regression analysis was created and, hazard risk ratios with 95 % confidence intervals were presented. If *P* values were <0.05, null hypotheses were rejected.

## Results

### General characteristics

Among the screened patients, 1065 patients had been undergone stress echocardiography for evaluation of cardiac ischemia and had normal findings. Among these patients, 243 patients were excluded due to having various cardiac diseases mentioned in the exclusion criteria. Four patients had been mislabeled as the indication for stress test was not for evaluation of possible ischemia. From the remaining 818 cases that were followed-up, 98 cases were dropped as they did not reach a minimum to two years from index stress study and additional 15 patients could not be reached for follow-up. Finally, data for 705 patients were recorded for the final analysis.

The mean age of patients at the time of the index stress echocardiography was 53.1 ± 11.6 years. Of the study population, 180 (25.5%) were male, and 525 (74.5%) were female. The prevalence of diabetes, hypertension, and dyslipidemia were 5.7%, 43.5%, and 14.2%, respectively. Among the study sample, 5.5% were smokers, and 7.5% had a family history of cardiovascular diseases. The mean body mass index (BMI) of patients was 27.6 ± 4.4 kg/m^2^. The most common clinical symptom was atypical chest pain, with a prevalence of 77.7%, followed by dyspnea on exertion, which was present in 12.6% of the study population. At the time of stress echocardiography, 38.7% of patients had no cardiac risk factors, 48.9% had one cardiac risk factor, 9.9% had two risk factors, and 2.5% had three or more cardiac risk factors. Among 705 patients, 575 (81.6%) had undergone ESE and 130 (18.4%) had undergone DSE.

### Study endpoints

The mean duration of follow-up was 55.7 ± 17.5 months (median: 58 months). During the follow-up period, MACE occurred in 35 (5.0%) of patients. There were 11 deaths (1.6%), 24 (3.4%) hospital admissions, 27 (3.8%) conventional coronary angiographic examinations, 3 (0.4%) cerebrovascular events, 7 (1.0%) non-fatal acute coronary syndromes and 8 (1.1%) percutaneous or surgical coronary revascularizations during the follow-up period. Among 27 patients who underwent a coronary angiographic examination, 9 (33.3%) had normal coronary arteries, and 4 (14.8%) had non-significant coronary artery stenosis. Additionally, 8 (29.6%), 4 (14.8%) and 2 (7.4%) patients had one-vessel, two-vessel and three-vessel coronary stenosis in the coronary angiographic examination. During the follow-up period, coronary CT angiography was done in two patients with normal/non-significant coronary stenosis in both patients (100%). Myocardial perfusion scanning was performed in four patients from whom three (75%) had significant ischemia, and one (25%) had a normal/non-significant result.

Table 1 shows the initial clinical symptom of patients and the risk of developing MACE during the follow-up period. Patients with dyspnea on exertion were more likely to experience MACE (31.4% vs. 11.7%, *P*=0.002), and patients with presenting symptom of atypical chest pain were less likely to experience MACE during the follow-up period (48.6% vs. 79.2%, *P* <0.001).

**Table 1:** Clinical/referring symptom of patients with negative stress echocardiography test and the risk of developing any event during the follow up period

	**Negative composite event**	**Positive composite event**	**P value**
Atypical chest pain	528 (79.2%)	17 (48.6%)	<0.001
Typical chest pain	5 (0.7%)	0 (0.0%)	-
Dyspnea on exertion	78 (11.7%)	11 (31.4%)	0.002
Palpitation	8 (1.2%)	2 (5.7%)	0.084
Asymptomatic	48 (7.2%)	5 (14.3%)	0.174

**Figure 1:**
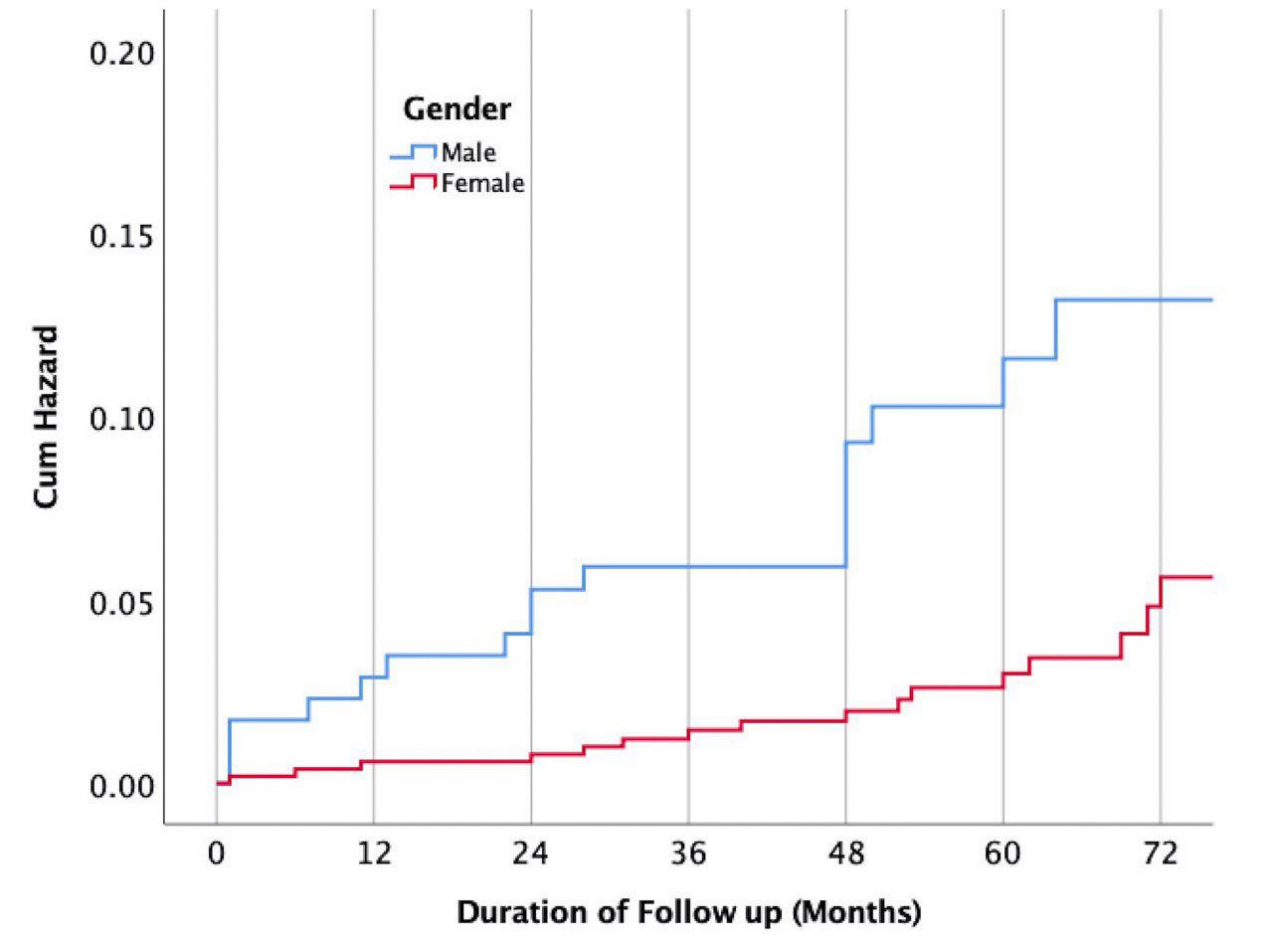
Kaplan-Meier survival curves (considering major cardiovascular adverse events as the endpoint) in patients with normal stress echocardiography stratified according to patient’s sex.

**Figure 2:**
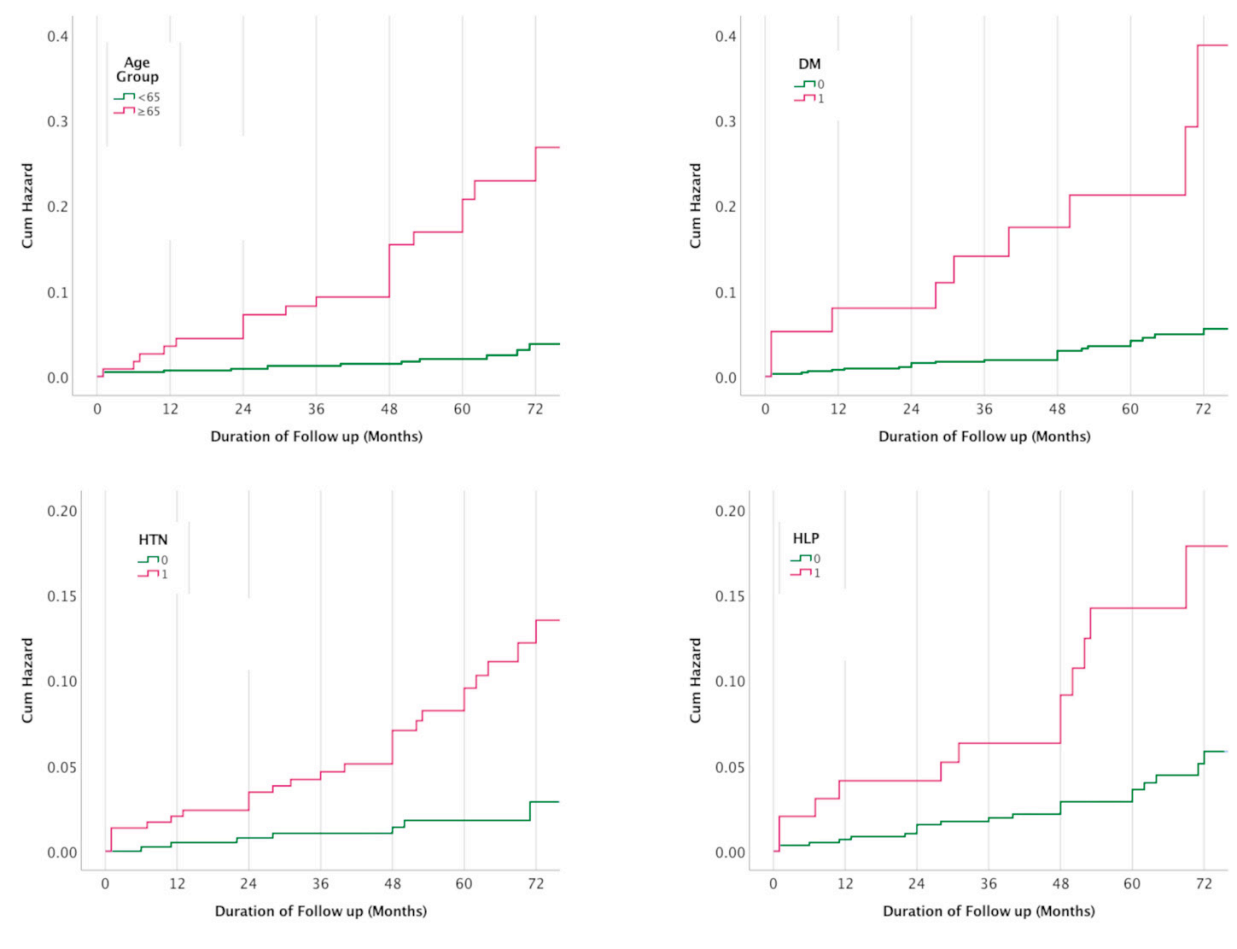
Kaplan-Meier survival curves (considering major cardiovascular adverse events as the endpoint) in patients with normal stress echocardiography stratified according to age group ≥5-years-old, diabetes, hypertension and hyperlipidemia.

**Figure 3:**
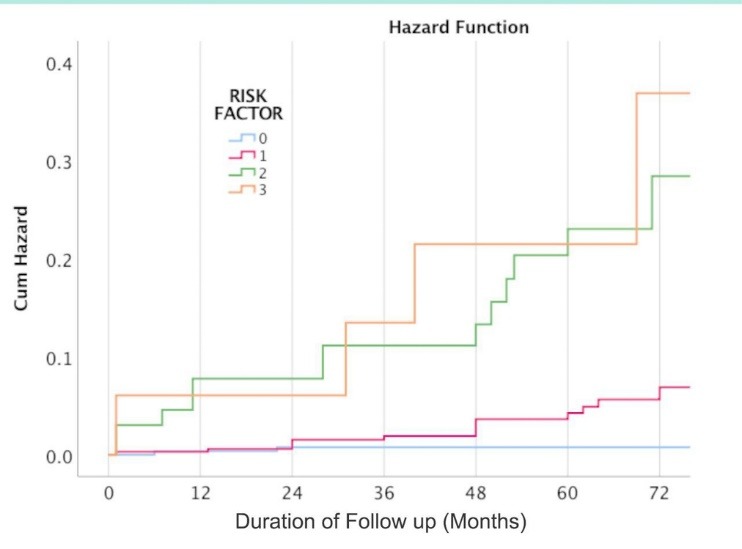
Kaplan-Meier survival curves (considering major cardiovascular adverse events as the endpoint) in patients with normal stress echocardiography stratified according to the number of cardiovascular risk factors.

Table 2 presents the impact of demographic and cardiac risk factors on developing MACE during the follow-up period. Females were significantly less likely to experience MACE in comparison to males (3.1% vs. 10.5%, RRR: 0.27, 95% CI: 0.13-0.53, *P* < 0.001) (Figure 1). Patients in the age group ≥65 years old were significantly more likely to experience MACE (17.9% vs. 2.4%, RRR: 8.94, 95% CI: 4.39-18.18, *P* < 0.001). Diabetics had significantly increased risk of MACE (22.5% vs. 3.9%, RRR: 7.11, 95% CI: 3.07-16.47, *P* < 0.001). Hypertensive patients had also higher risk of MACE (9.2% vs. 1.8%, RRR: 5.61, 95% CI: 2.42-13.03, *P* < 0.001). Similarly, patients with hyperlipidemia experienced more MACE in comparison to patients without the disease (12.0% vs. 3.8%, RRR: 3.44, 95% CI: 1.65-7.16, *P* = 0.002) (Figure 2). Meanwhile, smoking status and family history of cardiovascular diseases were not associated with MACE. Patients with a higher number of cardiovascular risk factors were significantly more likely to experience MACE (Figure 3). Among patients with three or more cardiovascular risk factors, 23.5% experienced MACE during the follow-up period. However, only 0.8% of patients without any cardiovascular risk factors at the time of stress echocardiography experienced MACE, later during the follow-up period.

**Table 2:** The prevalence of composite events according to the presence of cardiac risk factors

**Risk factors**		**MACE, No. (%)**	**Relative risk ratio**	***P*** ** value**
Sex	Male	19 (10.5)	0.27 (0.13-0.53)	<0.001
	Female	16 (3.1)		
Age group	< 65 years old	14 (2.4)	8.94 (4.39-18.18)	<0.001
	≥ 65 years old	21 (17.9)		
Diabetes mellitus	Non-diabetics	26 (3.9)	7.11 (3.07-16.47)	<0.001
	Diabetics	9 (22.5)		
Hypertension	Non-hypertensives	7 (1.8)	5.61 (2.42-13.03)	<0.001
	Hypertensives	28 (9.2)		
Dyslipidemia	Normal Lipid Profiles	23 (3.8)	3.44 (1.65-7.16)	0.002
	Hyperlipidemia	12 (12.0)		
Smoking status	Current smoker	32 (4.8)	1.65 (0.48-5.63)	0.435
	Non-smoker /quit > 6 months	3 (7.7)		
Family history	No	33 (5.1)	0.73 (0.17-3.14)	>0.999
	Yes	2 (3.8)		
Total number of risk factors	No risk factor	2 (0.8)		<0.001
	One risk factor	15 (4.5)		
	Two risk factors	14 (20.6)		
	Three or more risk factors	4 (23.5)		

**Figure 4:**
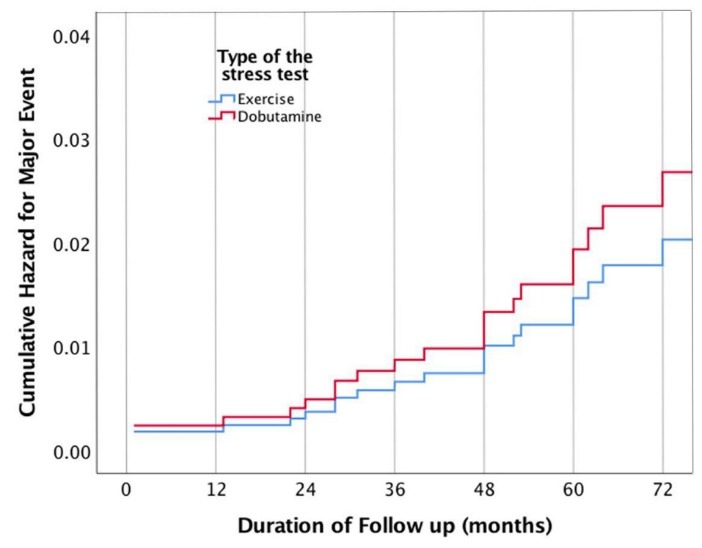
Kaplan-Meier survival curves (considering major cardiovascular adverse events as the endpoint) in patients with normal stress echocardiography stratified according to the type of stress echocardiography (exercise vs. dobutamine stress echocardiography)

Interestingly, patients who underwent DSE had significantly higher MACE in comparison to patients who underwent ESE (10.8% vs. 3.0%, *P* = 0.001) (Figure 4). The NPV as calculated by the number of true negative test (number of the patients with no event) divided by the total number of negative studies was significantly less for DSE than those for ESE (*P* = 0.001). From a total of 130 negative DSE exam, no event occurred in 116 patients (NPV = 89.2%), while 555 patients from 575 patients with negative ESE did not develop any event during the follow-up period (NPV = 96.5%).

The univariate comparison of echocardiographic findings patients with and those without MACE is shown in Table 3. The mean HR_max_ was significantly lower in patients with MACE. Patients in the MACE group had a trend toward a higher SBP_max_, but this was not statistically significant (*P* = 0.071). Maximum diastolic and mean arterial pressures were similar in two groups. Baseline left ventricular ejection fraction and the percentage of change in ejection fraction were also similar in two groups. Systolic pulmonary artery pressure was significantly higher in patients of the MACE group (30.3 ± 7.2 mm Hg vs. 25.9 ± 4.9, *P* = 0.002). The ratio of the trans-mitral early peak velocity (E) over early diastolic mitral annulus velocity (E’) (E/E’), were similar in two groups. In ESE subgroup, METS was significantly lower in the MACE group 9.6 ± 2.6 vs. 10.7 ± 2.3, *P* = 0.041). In this subgroup of patients, RPP_max_ / METS activity was significantly higher in the MACE group (329 ±146 vs. 258 ± 74 cmHg*bpm, *P* < 0.001) (Table 3).

**Table 3:** Comparison of echocardiographic variables in patients with and those without composite cardiac events during follow-up period

	**Negative Event**	**Positive Event**	***P*** ** value**
	**Mean (SD)**	**Mean (SD)**	
Peak heart rate (bpm)	158 (20)	147 (20)	0.001
Systolic blood pressure (mm Hg)	160 (25)	168 (29)	0.076
Diastolic blood pressure (mm Hg)	89 (14)	86 (16)	0.204
Mean Arterial pressure (mm Hg)	113 (16)	113(19)	0.903
Rate-pressure product-RPP_max_ (cmHg*HR)	2546 (537)	2498 (648)	0.631
Baseline ejection fraction (%)	55 (2)	55 (2)	0.543
Ejection fraction after stress (%)	66 (4)	65 (5)	0.116
Percentage of change in ejection fraction	19.9 (6.1)	18.3 (9.4)	0.216
Systolic pulmonary artery pressure (mm Hg)	26 (5)	30 (7)	0.002
E/E'	7.9 (2.2)	6.9 (2.8)	0.528
Metabolic equivalent of task (METS)*	10.7 (2.3)	9.6 (2.6)	0.041
RPP_max_ / METS activity*	258 (74)	329 (146)	<0.001

*Calculated only in patients with a normal exercise stress echocardiography. E/E’: The ratio of the trans-mitral early peak velocity (E) over early diastolic mitral annulus velocity (E’).

Table 4 represents the results of Cox regression multivariate analysis of the risk factors for developing MACE in all study samples as well as in ESE subgroup and DSE subgroups individually. Considering all study sample, male sex, age group ≥65-year-old, diabetes, hypertension, and hyperlipidemia were independent predictors of MACE in multivariate Cox regression analysis. However, systolic pulmonary pressure and rate pressure product were not independent predictors of long-term composite cardiac events. In patients who underwent ESE, male sex, age ≥ 65-year-old and diabetes mellitus were independent predictors of long-term composite cardiac events. Higher systolic pulmonary blood pressure and RPP_max_/METS activity levels were both independent predictors of MACE (Table 4). Meanwhile in DSE subgroup, male sex, age ≥ 65-year-old and hyperlipidemia were only independent factors associated with developing adverse outcomes. However, systolic pulmonary artery pressure and RPP_max_ were not associated with development of MACE in this subgroup of patients. Due to the lower study sample of patients who underwent DSE, no patients in the MACE group had a family history of premature cardiovascular diseases, and none of the patients in the MACE group were current smokers. As a result, these variables were not suitable to include in multivariate regression analysis in this subgroup of patients.

**Table 4:** Cox multivariate regression analysis for composite cardiac events in all patients as well as according to the nature of the stress echocardiography performed

	**Regression coefficients**	**Standard error**	**Hazard ratio**	**95% CI**	***P*** ** value**
Female/male	-1.527	0.405	0.217	0.098	0.480	**< 0.001**
Age ≥ 65 year old	1.342	0.429	3.826	1.652	8.863	**0.002**
Diabetes mellitus	1.325	0.479	3.764	1.473	9.616	**0.006**
Current smoking	0.290	0.647	1.336	0.376	4.752	0.655
Hypertension	1.182	0.522	3.260	1.173	9.062	**0.023**
Hyperlipidemia	1.088	0.412	2.968	1.324	6.654	**0.008**
Family history	-0.309	0.803	0.735	0.152	3.544	0.701
RPP_max_ (cmHg*beats per minute)	0.000	0.000	1.000	1.000	1.000	0.415
Systolic pulmonary artery pressure	0.043	0.029	1.044	0.986	1.104	0.140
**Exercise Echocardiography Subgroup**						
Female/male	-1.637	0.589	0.195	0.061	0.617	**0.005**
Age ≥ 65 year old	1.803	0.707	6.069	1.518	24.271	**0.011**
Diabetes mellitus	2.864	0.799	17.527	3.663	83.854	**<0.001**
Current smoking	0.147	0.843	1.158	0.222	6.051	0.862
Hypertension	0.768	0.678	2.155	0.570	8.147	0.258
Hyperlipidemia	0.216	0.750	1.242	0.286	5.396	0.773
Family history	0.267	0.908	1.307	0.220	7.745	0.768
Systolic pulmonary artery pressure	0.122	0.043	1.130	1.039	1.229	**0.004**
RPP_max_ (cmHg*Beats per minute)/ METS activity	0.001	0.000	1.001	1.000	1.001	**0.004**
Dobutamine Echocardiography Subgroup						
Female/male	-1.895	0.612	0.150	0.045	0.499	**0.002**
Age ≥ 65 year old	1.734	0.653	5.662	1.575	20.349	**0.008**
Diabetes mellitus	0.675	0.719	1.964	0.480	8.034	0.348
Hypertension	1.973	1.048	7.189	0.921	56.124	0.060
Hyperlipidemia	1.316	0.564	3.728	1.234	11.269	**0.020**
Systolic pulmonary artery pressure	0.006	0.045	1.006	0.920	1.100	0.897
RPP_max_ (cmHg*Beats per min)	0.000	0.000	1.000	1.000	1.000	0.250

RPP_max_: Maximum Rate-Pressure product; METS: metabolic equivalent of tasks.

## Discussion

According to the findings of this study, in patients with a normal ESE/DSE, the event-free survival was shorter in men within the age group of ≥65-year-old who were diagnosed with diabetes mellitus, hypertension, and hyperlipidemia. Although the echocardiographic findings do not independently predict event-free survival in patients with a normal stress exam, the results can be influenced by the type of stress echocardiography examination. While higher systolic pulmonary artery pressure and RPP_max_/METS activity significantly decreased the event-free survival in patients with normal ESE after adjusting for cardiovascular risk factors, higher systolic pulmonary artery and pressure RPP_max_ were not independent predictors of lower event-free survival in DSE subgroup after adjusting for the preexisting cardiac risk factors in multivariate analysis.

Our study revealed a NPV of 89.2% for DSE, and a NPV of 96.5% for ESE in predicting the occurrence of MACE. In a meta-analysis by Metz et al, NPV for ESE was calculated as 98.4% over 33 months.^7^ In the setting of emergency room in patients with symptoms suggestive of CAD, Bedetti et al, found a NPV of 98.8% for pharmacological stress echocardiography.^15^ The NPV of DSE in women was 79% in a study by Rollan el al.^16^ In a report by Amici el al during a four year follow-up infarct-free NPV was 97% for pharmacologic stress echocardiography^17^ DSE was also reported to have a NPV 96% during a 6 month period.^18^ While our study confirms the high NPV normal ESE/DSE, the differences may represent the varying inclusion criteria, definition of adverse outcomes and duration of follow-up.

The prevalence of MACE was 17.9% in patients of the age group ≥ 65 years old in our study sample. We found that being in older age group was a predictor of MACE despite a normal stress exam. This finding was true in both DSE and ESE subgroups. Chaowalit et al studied the predictor of adverse outcomes in patients with a normal DSE and found age as a predictor of cardiac events and all-cause mortality in both univariate and multivariate analysis.^19^ Similarly, Chung et al found that the patients with a normal DSE or ESE who experienced cardiac events within one year were older. However, the comparison of patients regarding the adverse outcomes was not reported in a multivariate analysis.^20^ McCully et al investigated patients with normal ESE without a history of prior CAD and determined older age as an independent factor of adverse outcomes.^21^ Barbieri et al reported older age as the only predictor of composite adverse cardiac events in multivariate analysis of patients with normal ESE and no prior history of CAD, but age was not an independent factor when they did not consider hospitalization for acute heart failure or atrial fibrillation in their composite events. Dyslipidemia and workload were the only predictors of their primary end-point.^22^ As we excluded patients with a previous history of CAD and found age group ≥ 65 years old as an independent predictor of MACE after adjustment for cardiac risk factors and echocardiographic findings in ESE and DSE groups, negative results of stress echocardiography should be interpreted with caution in this particular age group.

The prevalence of MACE was 10.1% in males in comparison to 3.5% in females. Our results revealed male sex as an independent predictor of long-term MACE in both normal DSE and ESE subgroups. In the study of Chaowalit et al males with a normal DSE had higher long-term all-cause mortality, but cardiac events were similar in males and females.^19^ In a clinical trial, performed by Laiq et al male sex was associated with lower event-free survival.^23^

Velasco del Castillo et al studied patients with negative ESE and found male sex as a risk factor for future adverse events only in univariate analysis but not in multivariate analysis. It should be noted that their model included the European SCORE in which age and sex are already included^24^ In the study of McCully et al gender was not associated with adverse outcomes in patients with normal ESE.^21^ On the other hand, female sex was an independent predictor of long-term adverse events in the study by Al-Mallah et al in which they had included patients with normal ESE. However, their study did not exclude patients with prior CAD.^25^

While smoking status and family history of heart diseases were not associated with adverse outcomes, our findings indicated that diabetic patients had a 22.5% rate of developing MACE after a normal stress study. Both hypertension and hyperlipidemia were increased risk of MACE. The increased number of cardiovascular risk factors was associated with a higher risk of long-term MACE. In multivariate Cox regression analysis, diabetes was an independent predictor of MACE in patients of ESE subgroup. Hyperlipidemia was an independent predictor of MACE in DSE subgroup.

In the study of Kamalesh et al, diabetic and non-diabetic patients with a normal stress study were followed up for a mean duration of 25 months, and MACE was found to be significantly higher than non-diabetic patients. The patients with the previous CAD were not excluded in this study.^26^ The results of the report by Chung et al revealed diabetes as a predictor of adverse outcomes in patients with normal ESE and no history of CAD.^20^ However, diabetes was not independently predicted adverse outcome in the study of Barbieri et al on patients with normal ESE ^22^ as well as in the study of Al-Mallah et al^25^ and McCully et al.^21^ Unlike the results of our study regarding the subgroup of patients with normal DSE, Chaowalit et al found diabetes as an independent predictor of adverse outcomes. It should be noted that unlike our study, patients who did not reach the target heart rate were included in their study sample.^19^

Cortigiani et al studied the prognostic value of stress echocardiography in a large cohort of hypertensive and normotensive patients with known or suspected CAD. Their findings showed similar rates of revascularization after a normal study, in patients with and those without hypertension. However, hypertension was associated with a significantly increased risk of adverse outcomes in patients aged younger than 65 years with a normal test result.^9^ In the study of Chaowalit et al among the cardiovascular risk factors age, male sex and, diabetes mellitus were predictors of all-cause mortality in patients with normal DSE. Age, diabetes, and hypertension were the independent predictors of cardiac events.^19^ Mccully et al found hypertension and diabetes as a predictor of follow-up cardiac events in univariate but not multivariate analysis.^21^ Marwick et al determined diabetes and hypertension as predictors of cardiac death in the follow-up of patients with normal DSE.^27^ In the study of Al-Mallah et al on patients with normal ESE, hypertension, diabetes, and smoking were not independent factors of long-term MACE in multivariate Cox regression analysis.^25^ In the study of Velasco del Castillo et al. on patients without ischemia in ESE, smoking, dyslipidemia, hypertension, and diabetes were associated with a higher risk of adverse events in univariate analysis but lost their significance in multivariate analysis after adjustment for other stress echocardiography variables.^24^

In patients with normal coronary arteries, coronary vascular dysfunction is a significant predictor of increased cardiovascular adverse events.^28^ Microvascular changes in diabetic patients which may be present in the absence of coronary artery involvement or left ventricular dysfunction, is shown to predict a poor prognosis as well.^29^ In hypertensive patients, presence of left ventricular hypertrophy is an indicator of poor prognosis.^30^ On the other hand, in patients with non-obstructive CAD, left ventricular hypertrophy was proved to be associated with the presence and extent of myocardial ischemia determined by myocardial contrast echocardiography.^31^ As a result, the presence of microvascular coronary dysfunction, which is not necessarily induce wall motion abnormality, may contribute to adverse cardiac events in patients with normal stress echocardiography.

RPP_max_ shows the metabolic demand of myocardium, have been studied in a few studied in this setting.^32^ We found no significant differences in RPP_max_ in our study sample. METS which was available in patients with ESE were significantly lower in patients with higher risk of MACE. For a better assessment of RPP, we calculated the RPP_max_ over METS. RPP_max_ / METS activity was revealed as an independent predictor of MACE in patients with normal ESE after adjusting for age, sex, and other cardiovascular risk factors. Peteiro et al reported lower peak double product in patients with cardiac events during the follow-up period after undergoing ESE.^33^ Van der Sijde et al found increased RPP_max_ as a univariate but not multivariate predictor of long-term cardiac death in a sample of patients with both positive and negative DSE.^34^ However, in the study of Mccully et al RPP_max_ was not associated with the adverse outcome in patients with normal ESE^21^. In the study of Marwick et al lower RPP_max_ was associated with higher adverse events in patients with normal DSE.^27^ Velasco del Castillo et al, found decreased RPP_max_ as a predictor of cardiac events after a normal ESE in univariate but not multivariate analysis.^24^

Stress induced pulmonary artery pressure increase may be attributable to increased filling pressure of left ventricle as well as pulmonary resistance. Misra et al demonstrated that 11.7% of patients, who underwent clinically indicated ESE, had elevated pulmonary artery pressures with exercise. The increased stress induced pulmonary pressure was indicative of abnormal right heart hemodynamics in catheterization.^35^ In patients with preserved left ventricular ejection fraction, the increased risk for adverse cardiac events was observed in those with exercise-induced pulmonary hypertension with an increase in estimated LV filling pressure during exercise.^36^ While there is limited data regarding the role of pulmonary artery pressure measured by ESE, in this study we found the exercise induced systolic pulmonary artery pressure as an independent predictor of future MACE, in patients with normal ESE. While we did not directly examine the presence of pulmonary hypertension, our results may indicate the presence of other underlying disorders that predispose patients to higher risk of adverse events despite a normal ESE. However, after adjustment for other risk factors, this association was not present in patients who underwent DSE. It should be noted that estimation of systolic pulmonary artery pressure after dobutamine administration may differ from the estimation derived by ESE.^37^ Generally evaluation of diastolic function by DSE is not recommended. There is only a report in which the effect of DSE on left ventricular filling pattern was investigated in patients with ischemic cardiomyopathy. The results of this report revealed that persistent restrictive left ventricular filling pattern in response to DSE was associated with a poor long-term outcome.^38^

## Limitations

This study is a single center cohort study. Also, the study sample included patients, referred for evaluation of CAD to our tertiary level echocardiology laboratory, which increases the possibility for the referral bias. Most of our patients had undergone ESE, and the sample size was smaller in the subgroup of patients who had undergone DSE. Further studies with a higher number of patients with a normal DSE may yield all potential hemodynamic and echocardiographic prognostic factors in this patient population with a lower general health level.

## Funding

This research received no specific grant from any funding agency in the public, commercial, or not-for-profit sectors.

## Competing interests

The authors declare that there is no conflict of interest.

## Ethical approval

All procedures were in accordance with the ethical standards of the responsible committee on human experimentation of our academic institution and with the Helsinki Declaration of 1975, as revised in 2000. All patients signed a privacy of health information authorization for research while they signed an informed consent for the procedure. Patient privacy was maintained in all study process.
